# Hotel Dieu

**Published:** 1829-12

**Authors:** 


### HOSPITAL REPORTS.

### HOTEL DIEU.

### *Fracture of the Spine. Symptoms of Compression of the Spinal Marrow. Cure.*

INSTANCES of complete cure after fracture of the vertebral column are uncommon. The following case was recently admitted at the Hotel Dieu.

A strong healthy man, æt. twenty-eight, had fallen from the second floor of a house, and fractured the spine, upon a level with the tenth dorsal vertebra. The nature of the injury had been ascertained previously to his admission, and he had been bled four times. Immediately after the accident, the patient was taken up in a state of insensibility; from which, however, he speedily recovered. Symptoms of compression of the spinal marrow, with paralysis of the left inferior extremity, occurred on the second day. The inflammatory symptoms were violent, and they had been opposed by the repeated abstraction of blood, as above mentioned. He was again bled on his admission, on the 3d of September.

4th.—Upon examination, a considerable projection of the last dorsal vertebra, forming a curvature of three inches, the convexity of which was towards the right side, was discovered. The injured parts were examined as tenderly as possible. The left inferior extremity was completely paralysed. It had lost its sensibility and power of motion. The right limb retained its powers, and also the bladder and rectum. The patient was placed in a horizontal position, the loins being supported by a square pillow. He was fixed in bed by a folded sheet placed over his chest, and fastened to the irons of the bedstead. During the night he became delirious and feverish, and a strait-waistcoat was put on. Fourteen ounces of blood were taken away in the morning, and twenty-five leeches were immediately applied in the course of the jugular veins. Strict diet.

5th.—Patient much calmer. He was cupped on each side of the injured part of the back.

7th.—The symptoms of cerebral disturbance have passed off. Pulse less frequent. Left lower extremity still paralysed; bladder and rectum unaffected. He was again cupped near the injury. Light diluents were ordered, and diet as before.

From this time the pulse gradually became natural, and the general condition of the patient encouraging. He was desired to remain perfectly quiet. Light food was allowed.

At the latter end of September, he had almost entirely regained the sensibility of the paralysed limb. The power of moving it was more slowly restored.

October 14th.—He can now move the left limb nearly as well as the right. Sensibility quite restored. When he is placed in a horizontal position, he cannot raise the left heel to a great height. He complains also of some pain in the right hip-joint, but he had experienced this frequently before the accident. The projection of the dorsal vertebra is still evident, but is much diminished: it is only perceptible in the median line.

At the time the case was reported, the patient had not been permitted to leave his bed, although he was very desirous of doing so. His perfect recovery was considered certain.—*La Clinique.*

